# Detailed Protocol for Segmentation and Quantification of Overlapping Prospore Membranes using DeMemSeg

**DOI:** 10.21769/BioProtoc.5520

**Published:** 2025-12-05

**Authors:** Shodai Taguchi, Keita Chagi, Hiroki Kawai, Kenji Irie, Yasuyuki Suda

**Affiliations:** 1 Ph.D. Program in Humanics, School of Integrative and Global Majors, University of Tsukuba, Tsukuba, Ibaraki, Japan; 2 Laboratory of Molecular Cell Biology, Institute of Medicine, University of Tsukuba, Tsukuba, Ibaraki, Japan; 3 Bioinformatics Laboratory, Institute of Medicine, University of Tsukuba, Tsukuba, Ibaraki, Japan; 4 Research and Development Department, LPIXEL Inc., Chiyoda, Tokyo, Japan

**Keywords:** Deep learning, Instance segmentation, Mask R-CNN, Overlapping objects, Quantitative image analysis, Microscopy image processing, Yeast sporulation, Cellular morphology

## Abstract

Quantitative analysis of biological membrane morphology is essential for understanding fundamental cellular processes such as organelle biogenesis and remodeling. While manual annotation has been the standard for complex structures, it is laborious and subjective, and conventional automated methods often fail to accurately delineate overlapping objects in 2D projected microscopy images. This protocol provides a complete, step-by-step workflow for the quantitative analysis of overlapping prospore membranes (PSMs) in sporulating yeast. The procedure details the synchronous induction of sporulation, acquisition of 3D fluorescence images and their conversion to 2D maximum intensity projections (MIPs), and the generation of a custom-annotated dataset using a semi-automated pipeline. Finally, it outlines the training and application of our mask R-CNN-based model, DeMemSeg, for high-fidelity instance segmentation and the subsequent extraction of morphological parameters. The primary advantage of this protocol is its ability to enable accurate and reproducible segmentation of individual, overlapping membrane structures from widely used 2D MIP images. This framework offers an objective, efficient, and scalable solution for the detailed quantitative analysis of complex membrane morphologies.

Key features

• Provides a mask R-CNN-based pipeline to accurately segment individual, overlapping membrane structures resulting from 2D maximum intensity projections of 3D image stacks.

• Optimized for quantifying the dynamic morphology of yeast prospore membranes (PSMs), a key model system for studying de novo membrane biogenesis.

• Presents a complete workflow from single-cell isolation using a custom CellPose model to detailed manual annotation for creating high-quality training datasets.

• Enables robust quantitative phenotyping by extracting morphological parameters (e.g., length, roundness) to distinguish subtle differences between wild-type and mutant strains.

## Graphical overview


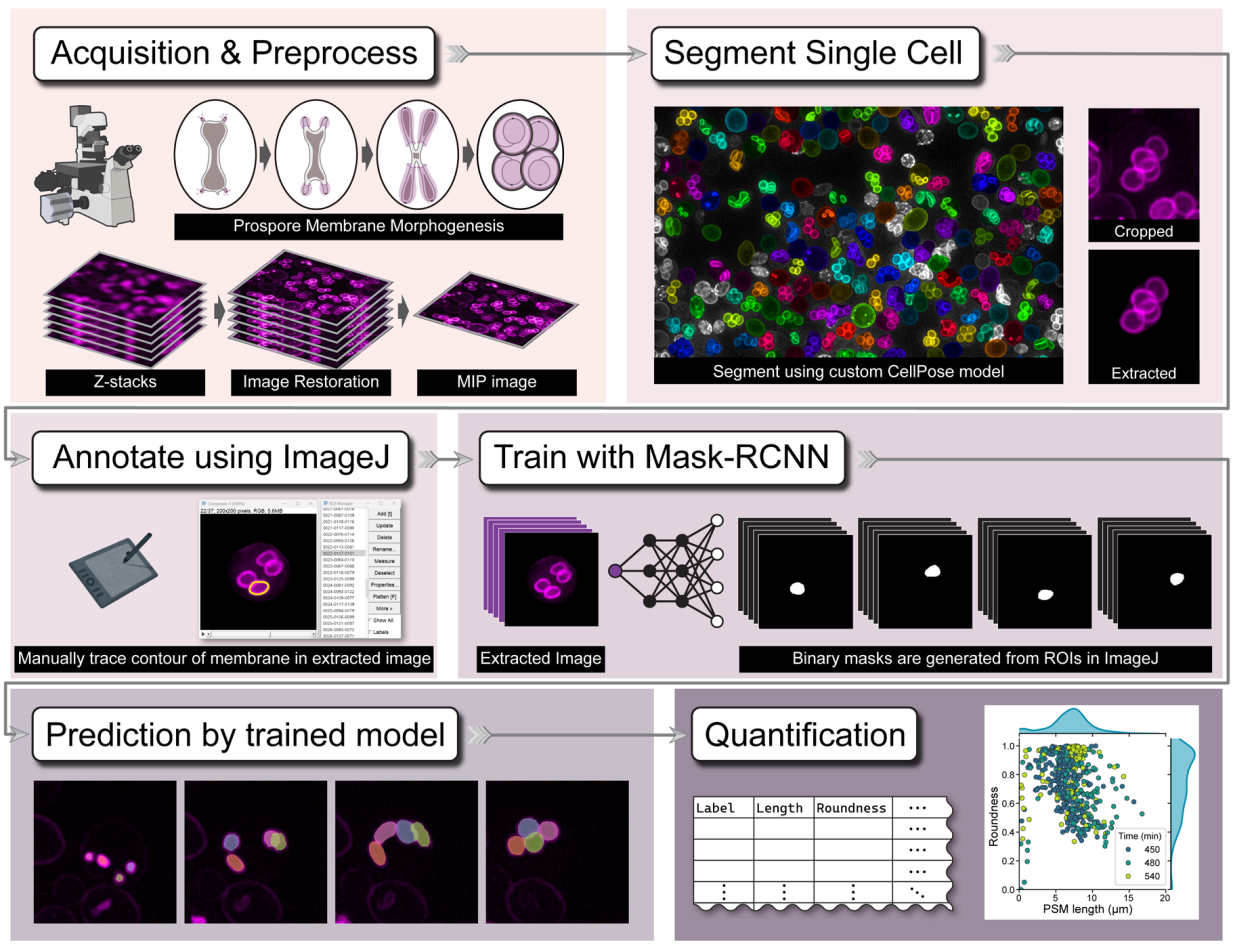


**Graphical overview of the DeMemSeg pipeline for automated segmentation and quantitative analysis**

## Background

Understanding fundamental cellular processes like organelle biogenesis, remodeling, and trafficking often requires quantitative analysis of biological membranes. This involves measuring key morphological features—such as perimeter and roundness—to describe their geometric shape. A key challenge in this field is the accurate segmentation of individual membrane structures from microscopy images, which is often a prerequisite for obtaining reliable quantitative data. The budding yeast prospore membrane (PSM), a model for de novo membrane formation, presents a typical challenge where dynamically growing membranes frequently overlap when visualized in 2D maximum intensity projection (MIP) images.

Historically, segmenting such complex structures has relied on meticulous manual annotation. While often considered the gold standard for accuracy, this method is extremely time-consuming, laborious, and prone to inter-observer variability, making it impractical for large-scale studies. Traditional automated methods based on intensity thresholding [1] or edge detection [2] often fail to accurately delineate boundaries in images with low contrast or, critically, to separate overlapping instances. The advent of generalist deep learning tools, most notably CellPose [3] and ilastik [4], has revolutionized biological image analysis by enabling robust, automated segmentation of individual cells, thereby greatly facilitating single-cell image analysis from dense populations. However, while these tools are highly effective for delineating whole-cell boundaries, their utility is often limited when analyzing more detailed and complex intracellular structures. Specifically, they frequently struggle to resolve extensively overlapping subcellular components like the PSMs within a single cell. Similarly, while large-scale foundation models like the Segment Anything Model (SAM 2) [5] show impressive zero-shot capabilities, our findings indicate they may not achieve the instance-level precision required for resolving the specific, artificial overlaps created by MIP imaging without specialized prompting or fine-tuning.

The DeMemSeg protocol presented here offers several advantages over these existing methods for this specific task. Its primary strength lies in the custom-trained mask R-CNN model, which is specifically optimized to recognize and separate overlapping PSMs in 2D MIP images with high fidelity, a capability where generalist models may falter. By providing a complete, automated workflow from single-cell isolation to instance segmentation, it dramatically improves efficiency and reproducibility compared to purely manual methods. The protocol's utility is demonstrated by its ability to yield quantitative data statistically comparable to manual annotation and to successfully characterize the complex morphological phenotype of an untrained mutant strain. The main limitation of this protocol is its supervised nature, requiring the upfront, labor-intensive creation of a high-quality annotated dataset. Additionally, as a 2D-based analysis, it cannot resolve the true 3D spatial relationships between structures, a general limitation of MIP image analysis.

Beyond yeast PSMs, the principles of this protocol are broadly applicable. The workflow of using a general cell segmentation model (like CellPose) for preprocessing and isolation, followed by training a specialized instance segmentation model (like mask R-CNN) on cropped single-cell images, can be adapted to analyze other challenging, overlapping subcellular structures, such as mitochondrial networks, the Golgi apparatus, or endoplasmic reticulum subdomains in various cell types. It is particularly well-suited for quantitative phenotyping in genetic or chemical screens where precise morphological measurements are required.

## Materials and reagents

**Biological materials**

1. *Saccharomyces cerevisiae* strains, SK1 background [6]

a. WT strain, YSIY1218

*MAT* **a**/*MAT*α *his3*Δ*SK/his3*Δ*SK ura3/ura3::pRS306-mCherry-Spo20^51–91^ trp1::hisG/trp1::hisG leu2/leu2 arg4-NspI/ARG4 lys2/lys2 ho*∆::*LYS2/ho*∆::*LYS2 rme1::LEU2/RME1 AUR1::P_ACT1_-LexA-ER-haVP16::AUR1-C/AUR1::P_ACT1_-LexA-ER-haVP16::AUR1-C*

*NDT80::hphNT1::P4×lexA-9×Myc-NDT80 /NDT80::hphNT1::P4×lexA-9×Myc-NDT80*

b. *gip1*∆ strain, YSIY793

*MAT* **a**/*MAT*α *his3*Δ*SK/his3*Δ*SK ura3/ura3::pRS306-mCherry-Spo20^51–91^ trp1::hisG/trp1::hisG leu2/leu2 arg4-NspI/ARG4 lys2/lys2 ho*∆:*:LYS2/ho*∆:*:LYS2 rme1::LEU2/RME1*

*AUR1::P_ACT1_-LexA-ER-haVP16::AUR1-C/AUR1::P_ACT1_-LexA-ER-haVP16::AUR1-CNDT80::hphNT1::P4×lexA-9×Myc-NDT80 /NDT80::hphNT1::P4×lexA-9×Myc-NDT80 gip1*∆::*kanMX6/gip1*∆::*kanMX6*

2. The plasmid used in this study [6] was pRS306-mCherry-Spo20^51–91^ *URA3, integration, pTEF1-mCherry-Spo20^51–91^*

**Reagents**

1. Yeast nitrogen base without amino acids (Difco YNB, catalog number: 291940)

2. D(+)-glucose (FUJIFILM Wako Pure Chemical Corporation, catalog number: 045-31167)

3. STAR agar L-grade 01 (Rikaken Co., catalog number: RSU-AL01-500G)

4. Bacto peptone (Thermo Fisher Scientific, catalog number: 211677)

5. Bacto yeast extract (Thermo Fisher Scientific, catalog number: 212750)

6. Adenine (6-Aminopurine) (FUJIFILM Wako Pure Chemical Corporation, catalog number: 012-11512)

7. Potassium acetate (FUJIFILM Wako Pure Chemical Corporation, catalog number: 167–3185)

8. β-Estradiol (Sigma-Aldrich, catalog number: E8875)

9. Amino acid mixture (five amino acid drop-out: arginine, leucine, uracil, histidine, tryptophan) (mixed manually in our lab)

**Solutions**

1. SD-RLU (see Recipes)

2. YPD medium (see Recipes)

3. YPA medium (see Recipes)

4. Sporulation medium (see Recipes)

5. Amino acid mixture (see Recipes)

6. 2 mM β-Estradiol (see Recipes)

**Recipes**

**1. SD-RLU**

**Table:** 

Reagent	Final concentration	Quantity or volume
Yeast nitrogen base without amino acids	6.7 g/L	3.35 g
Amino acid mixture	1 g/L	0.5 g
L-Histidine	0.16 g/L	0.08 g
L-Tryptophan	0.16 g/L	0.08 g
20% D(+)-glucose solution	2% D(+)-glucose	50 mL
Deionized water	n/a	450 mL
Total	n/a	500 mL

a. Dissolve all ingredients except 20% D(+)-glucose solution in 450 mL of deionized water.

b. Adjust the pH to 6.5 if necessary and then autoclave.

c. Allow medium to cool to ~55 °C and then add 50 mL of 20% dextrose (glucose) solution to 2% final concentration.

d. For making plates, add STAR agar L-grade 01 (2% at final concentration) before autoclaving.

**2. YPD medium**

**Table:** 

Reagent	Final concentration	Quantity or volume
Bacto yeast extract	10 g/L	5 g
Bacto peptone	20 g/L	10 g
Adenine	0.03 g	0.015 g
20% D(+)-glucose solution	2% D(+)-glucose	50 mL
Deionized water	n/a	450 mL
Total	n/a	500 mL

a. Dissolve all ingredients except 20% D(+)-glucose solution in 450 mL of deionized water and then autoclave.

b. Allow medium to cool to ~55 °C and add 50 mL of 20% dextrose (glucose) solution to 2% final concentration.

c. For making plates, add STAR agar L-grade 01 (2% at final concentration) before autoclaving.

**3. YPA medium**

**Table:** 

Reagent	Final concentration	Quantity or volume
Bacto yeast extract	10 g/L	5 g
Bacto peptone	20 g/L	10 g
Adenine	0.03 g	0.015 g
20% potassium acetate solution	2% potassium acetate	50 mL
Deionized water	n/a	450 mL
Total	n/a	500 mL

a. Dissolve all ingredients except the potassium acetate solution in 450 mL of deionized water and then autoclave.

b. Allow medium to cool to ~55 °C and add 50 mL of 20% potassium acetate solution to 2% final concentration.

**4. Sporulation medium**

**Table:** 

Reagent	Final concentration	Quantity or volume
20% potassium acetate solution	2% potassium acetate	50 mL
Deionized water	n/a	450 mL
Total	n/a	500 mL

a. Dissolve all ingredients and autoclave.

**5. Amino acid mixture**

**Table:** 

Reagent	Final concentration	Quantity or volume
Adenine (012-11512)	n/a	0.5 g
L-Alanine (010-01042)	n/a	2 g
L(+)-Arginine (017-04612)	n/a	(2 g)*
L-asparagine monohydrate (019-04812)	n/a	2 g
DL-aspartic acid (010-04842)	n/a	2 g
L-Cysteine hydrochloride monohydrate (033-05272)	n/a	2 g
L(+)-Glutamine (074-00522)	n/a	2 g
L-Glutamic acid hydrochloride (071-02092)	n/a	2 g
Glycine (073-00732)	n/a	2 g
L-Histidine (084-00682)	n/a	(2 g)*
*myo*-Inositol (092-00282)	n/a	2 g
L(+)-Isoleucine (121-00862)	n/a	2 g
L-Leucine (124-00852)	n/a	(10 g)*
L(+)-Lysine monohydrochloride (121-01462)	n/a	2 g
L-Methionine (133-01602)	n/a	2 g
*p*-Aminobenzoic acid (015-2332)	n/a	0.2 g
L(-)-Phenylalanine (161-01302)	n/a	2 g
L(-)-Proline (161-04602)	n/a	2 g
L-Serine (199-00402)	n/a	2 g
L(-)-Threonine (204-01322)	n/a	2 g
L-Tryptophan (204-03382)	n/a	(2 g)*
L-Tyrosine (202-03562)	n/a	2 g
Uracil (212-00062)	n/a	(2 g)*
L-Valine (228-00082)	n/a	2 g

*Quantities enclosed in parentheses indicate dropouts.

All amino acids are from FUJIFILM Wako Pure Chemical Corporation.

**6. 2 mM β-Estradiol**

**Table:** 

Reagent	Final concentration	Quantity or volume
β-Estradiol	2 mM	n/a
Ethanol	n/a	

a. Dissolve β-Estradiol in 100% ethanol to a final concentration of 2 mM.

b. Aliquot into 1.5 mL tubes and store at -30 °C.

**Laboratory supplies**

1. Sterile Veritable Petri dishes (Shallow, catalog number: 36-3412)

2. 24 × 50 mm cover slip (Matsunami Glass Ind., Ltd., No.1, catalog number: C024501)

3. 18 × 18 mm cover slip (Matsunami Glass Ind., Ltd., No.1, catalog number: C218181)

4. 250 mL Erlenmeyer flask (Corning, catalog number: 431144)

5. Incubator (PHC Holdings Corporation, catalog number: MIR-154-PJ)

6. Shaking water bath (Personal-11, TAITEC CORPORATION, catalog number: 0069409-000)

7. Clean bench (PHC Holdings Corporation, catalog number: MCV-131BNF)

8. Vortex mixer (LMS Laboratory and Medical Supplies, Brigachtal, catalog number: VTX-3000L)

9. Low speed benchtop centrifuge (TOMY, catalog number: LC-200)

## Equipment

1. THUNDER Imager Live Cell system (Leica Microsystems; includes a DFC9000 GTC sCMOS camera)

2. Workstation for computation (HP, OMEN Desktop; equipped with an NVIDIA GeForce RTX 3060 Ti GPU)

3. iPad Air (5th generation) (Apple Inc., model: MM9C3J/A)

4. Apple Pencil (2nd generation) (Apple Inc., model: A2051)

## Software and datasets

1. CellPose 3.1.0 (Oct 30, 2024, BSD, free)

2. MMdetection, v3.3.0 (Jan 5, 2024, Apache, free)

3. Fiji/ImageJ, 2.16.0/1.54p (Feb 17, 2025, GPL, free)

4. spacedesk Driver, 2.1.43 free (non-commercial private license)

5. Docker Engine, 27.1.4 (Dec 17, 2024, Apache, free)

## Procedure

This protocol is divided into two main parts. Part A details the complete procedure used to develop and train the DeMemSeg model, serving as a reference for users who wish to create a similar custom model for their own biological structures. Part B provides a ready-to-use guide for segmenting yeast prospore membranes (PSMs) using our pretrained DeMemSeg model. All computational steps are performed on a workstation equipped with a suitable NVIDIA GPU (e.g., RTX 3060 Ti 8GB VRAM). The entire software environment is managed using Docker to ensure reproducibility.

We highly recommend using an integrated development environment (IDE) like Visual Studio Code (VS Code) with its Dev Containers or Docker extension. This allows you to directly edit files and run commands inside the container from a user-friendly interface.

**A. Development and training of the DeMemSeg model**

This section describes the step-by-step procedure followed to create the annotated dataset and train the DeMemSeg model for PSM segmentation. Researchers aiming to train a model for other structures can adapt this workflow. The scripts and detailed instructions for this process are available in a GitHub repository designed for this purpose: https://github.com/MolCellBiol-tsukuba/DeMemSeg-train .

**A1. Yeast culture and sporulation induction**

1. Prepare yeast cultures

a. Streak yeast strains from frozen stocks onto a YPD plate. Then, streak single colonies onto appropriate SD-RLU agar plates and incubate at 30 °C for 2–3 days.

b. Inoculate a single colony into 3 mL of liquid SD-RLU medium in a test tube and grow for 24 h at 30 °C with vigorous shaking at 200 rpm.

2. Induce synchronous sporulation

a. Dilute 800 μL of the overnight pre-culture into 15 mL of YPA liquid medium in a 250 mL Erlenmeyer flask and incubate for 16 h at 30 °C.

b. To harvest and wash the cells, transfer the entire YPA culture to a 50 mL conical tube and pellet by centrifugation at 2,150× *g* for 3 min at room temperature. After discarding the supernatant, rinse the original culture flask with 15 mL of sterile water to collect any remaining cells and use this rinse to resuspend the cell pellet. Wash the cells by repeating the centrifugation step. Finally, resuspend the resulting pellet in sporulation medium (2% KOAc) to an optical density (OD_600_) of 2.0 and transfer to a 250 mL flask to resume incubation.

c. After 6 h of vigorous shaking, add 2 mM β-Estradiol to a final concentration of 2 μM to induce *NDT80* expression for the synchronized induction of sporulation.

**A2. Image acquisition and preprocessing**

1. Prepare samples for microscopy

a. Collect 200–300 μL of the sporulating cell culture at each desired time point following induction (e.g., every 30 min).

b. Perform a brief centrifugation (spin down) at a low speed (e.g., 500× *g* for 10 s) to form a soft cell pellet.

c. Gently resuspend the cell pellet by pipetting at the bottom of the tube.

d. Place approximately 5 μL of this concentrated cell suspension onto a 24 × 50 mm coverslip.

e. Gently place an 18 × 18 mm coverslip on top of the cell suspension to create a *sandwich*, spreading the cells between the two glass surfaces.

f. Absorb excess liquid by lightly pressing a Kimwipe on the top coverslip.

*Note: Our sample preparation method helps to gently immobilize and flatten the yeast cells into a monolayer. This approach improves image quality by bringing more cells into a single focal plane. Since the cells are not firmly embedded, this method is also well-suited for capturing snapshots of cellular states at specific time points. If you have a different preparation method, you can certainly use your standard procedure. After sample preparation, acquire images immediately using a microscope.*

2. Acquire Z-stack images

a. Place the prepared coverslip sandwich onto a microscope slide for imaging.

b. Using the Leica THUNDER Imager system with a HC PL APO 100×/1.40 OIL objective, acquire 3D Z-stacks (40–50 slices, 0.21 μm intervals) of the cells with the mCherry-labeled PSMs (mCherry-Spo20^51–91^).

3. Preprocess raw images

a. To reduce haze in the raw 3D Z-stack images, apply an image restoration or deconvolution method. For example, use the *Small Volume Computational Clearing* function in the Leica software or an equivalent image restoration function available on other microscope systems.

b. Generate 2D images by applying a maximum intensity projection (MIP) to the cleared Z-stacks. Export these 2D MIP images as TIF files.

**Tip:** Data organization and file naming: We recommend establishing a consistent file and folder naming convention. Create a project folder for each experiment that includes the strain name or condition (e.g., Project001_YSIY1218). When exporting images, embed key metadata into the filename. For example, manually rename the software-generated file Series002 to Series002_420min to indicate the imaging time point. The Leica software will automatically append its processing suffix (e.g., _Lng_SVCC_Processed001), resulting in a descriptive final filename like Series002_420min_Lng_SVCC_Processed

001.tif. This practice allows filenames to serve as self-contained metadata that can be used for sorting and analysis later.

**A3. Custom training dataset construction**

1. Create a custom CellPose model for preprocessing

a. Install CellPose with its graphical user interface (GUI). To manually create annotations, a separate local installation of CellPose is required, as the primary DeMemSeg Docker environment is configured for command-line execution and does not support the GUI. Follow the official installation instructions available at https://cellpose.readthedocs.io/en/latest/installation.html.

b. Prepare training data via manual annotation. Launch the local CellPose GUI and import a representative subset of your 2D MIP images. Use the drawing tools to manually trace the boundaries of individual cells. For this training set, it is critical to only annotate cells that are complete (not cut off by the image border) and alive. Live cells can be distinguished from dead cells by their distinct PSM fluorescence, whereas dead cells often exhibit a diffuse, whole-cell signal; exclude these dead and incomplete cells from your annotations.

c. Train the custom CellPose model. Within the CellPose GUI, initiate model training using your newly created annotations. Configure the training with the following parameters: a learning rate of 0.1, weight decay of 0.0001, and train for 10,000 epochs. Once training is complete, export the resulting custom model file for use in the main DeMemSeg pipeline.

**Tip:** For detailed, step-by-step technical instructions on how to train a custom model in CellPose 2.0, refer to established online guides such as the one found at https://mouseland.github.io/research/posts/cellpose2.html.

**Figure 1.**
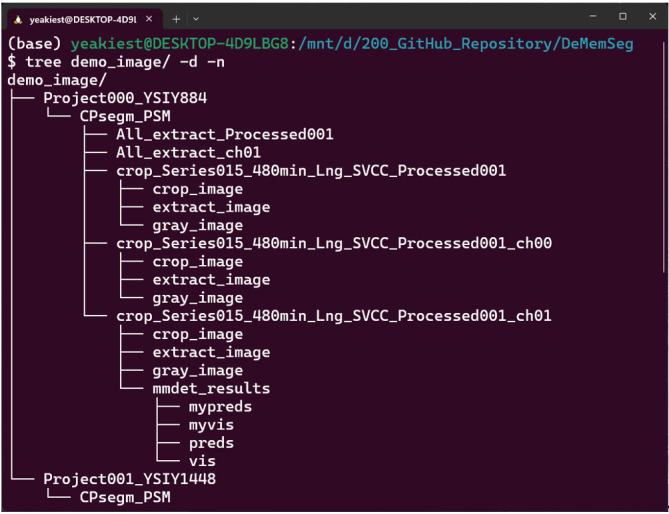
Example of the output directory structure. An example of the directory hierarchy created after running the CellPose-based single-cell extraction step. A main output directory, CPsegm_PSM, is generated within each project directory. Inside, results for each source image are organized into individual sub-directories. These contain the crop_image (a simple crop around the cell), the extract_image (an isolated single cell based on the segmentation mask), and its grayscale version, gray_image.

2. Isolate and process single-cell images

a. Apply the custom-trained CellPose model from the previous step to your full set of MIP images. This will automatically segment all valid single cells according to the learned criteria.

b. For each segmented cell, crop a 200 × 200 pixel region centered on its centroid. This process generates several image types for each cell, which are saved to separate folders for subsequent steps. crop_image: The raw 200 × 200 pixel region cropped directly from the multi-channel MIP image. extract_image: The crop_image with the corresponding CellPose mask applied to blank out any neighboring cells or background, isolating the single target cell. gray_image: A grayscale version of the extract_image (Figure 1).

**Figure 2.**
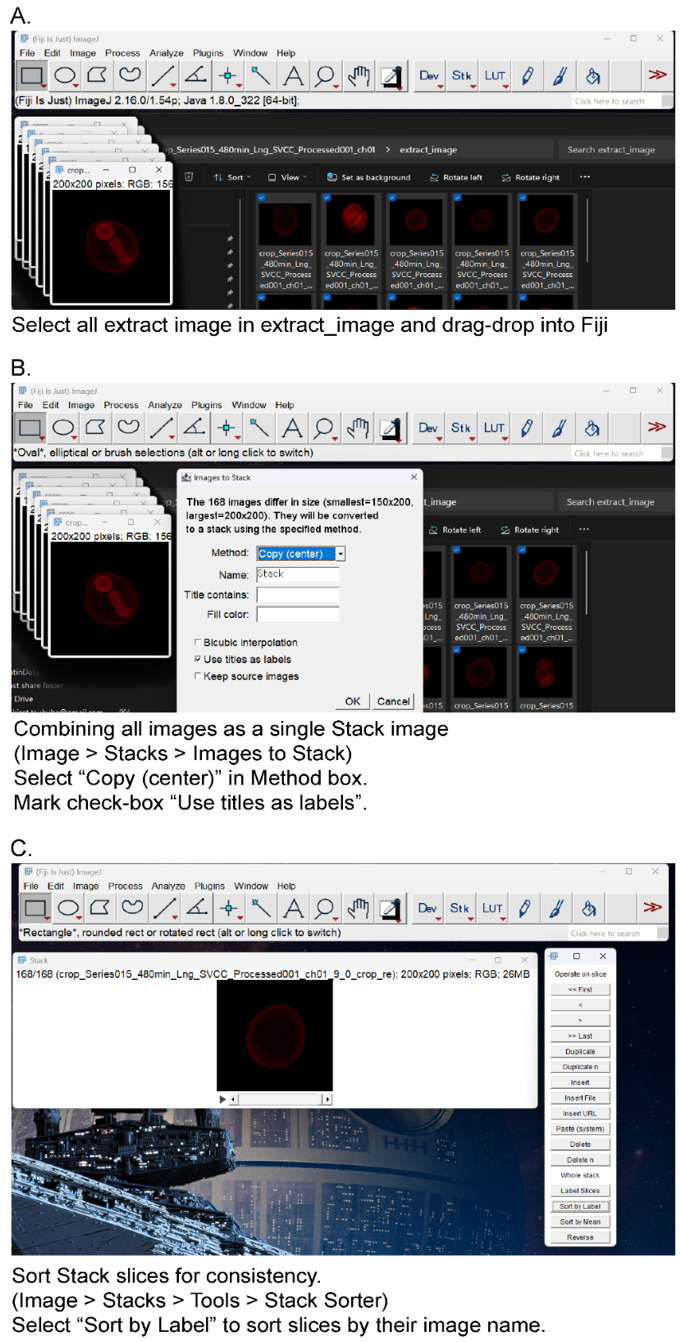
Create a stack image from cropped images. (A) Select all extract images in “extract_image” directory and drag/drop to Fiji/ImageJ. (B) From the menu bar, select *Image* > *Stacks* > *Images to Stack*. Mark the checkbox “Use title as labels” and select *Copy (center)* in the *Method* box. (C) Sort stack slices for consistency. From the menu bar, select *Image* > *Stacks* > *Tools* > *Stack Sorter*. Select “Sort by Label” in the *Stack Sorter* window. The slices are sorted by their image name.

**Figure 3.**
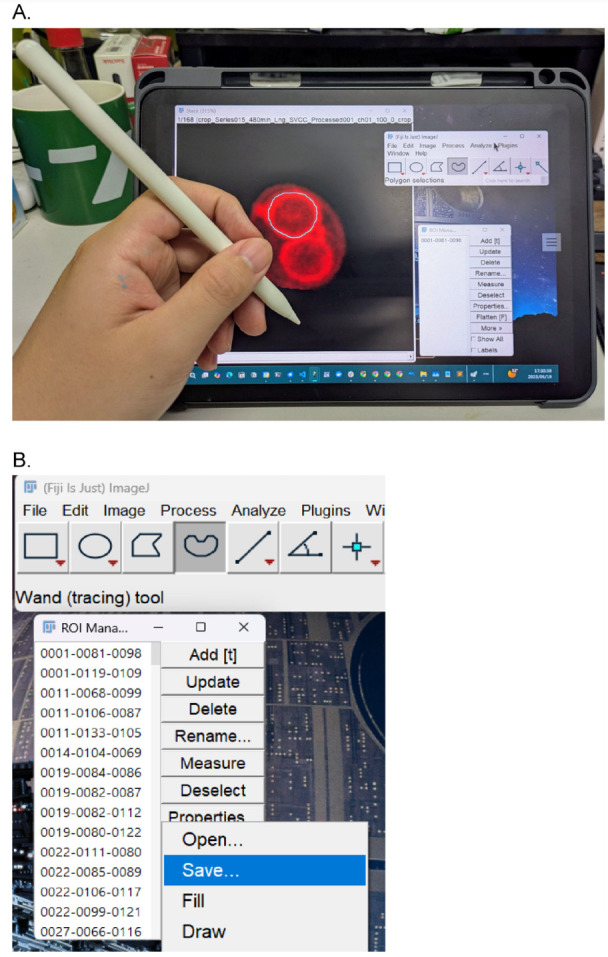
Manual annotation setup. (A) Use an iPad as a sub-monitor connected to a PC running the Fiji app. Tracing prospore membranes (PSMs) with an Apple Pencil. Click *Add* to register the PSM as a region of interest (ROI). (B) Save all ROIs (select all ROIs, click *More* > *Save*).

3. Manually annotate target structures (PSMs):

a. Create a stack image with image label as metadata in Fiji/ImageJ (Figure 2).

b. Manual annotation setup (Figure 3): For precise tracing, establish a desktop connection between your PC and an iPad using software such as Spacedesk. This allows for the use of an Apple Pencil as a drawing input.

c. Open images in Fiji/ImageJ. For each cell to be annotated, open the corresponding single-cell image from the extract_image folder. This image will serve as the base for creating a stack of regions of interest (ROIs).

d. Trace PSM boundaries. Using the *Freehand selection* tool, carefully trace the contour of a single PSM. Add this selection to the ROI Manager. Repeat this process for every individual PSM within the image, saving each as a separate ROI. Save the annotation files. For each annotated image, create a dedicated folder. Within this folder, save the set of ROIs from the ROI Manager as a .zip file (e.g., Stack.zip). It is also recommended to save the original stack image as a .tif file (e.g., Stack.tif) in the same folder for quality control.

4. Finalize the dataset for training

a. Collect a sufficient number of ROIs. Proceed to the next step after you have annotated a sufficient number of individual PSMs. For example, in our original work, we used approximately 7,000 ROIs to achieve the reported performance [7].

b. Convert ROIs to binary masks. The ROIs saved from ImageJ's ROI Manager as a .zip file (as described in section A3.3) must be converted into binary mask images. To do this, run the provided rois2mask.py script. This script processes the .zip file and generates a separate binary mask image for each ROI, where the annotated region has a pixel value of 1 and the background is 0.

c. Split the dataset. Next, randomly divide the entire set of image-mask pairs into training, validation, and testing sets using an 8:1:1 ratio. It is critical to ensure that each original image and its corresponding mask remain paired during this process. d. Create the COCO dataset. Finally, generate the annotation file in the COCO format, which is required by the MMdetection framework. Run the provided create_coco_annotations.py script, which compiles all annotations, including the image filenames and their corresponding masks in RLE format, into a single JSON file. This file is the final dataset used to train the model.

**A4. DeMemSeg model training**

1. Set up the training environment

a. Use the provided Dockerfile (available in the repository) to build and run a container with all necessary dependencies (MMdetection, PyTorch, etc.).

2. Configure and run training

a. Modify the model configuration file (e.g., configs/dememseg_config.py) to point to your new COCO-formatted dataset. This typically involves updating the data_root and annotation file path variables.

b. Adjust hyperparameters in the configuration file if necessary (e.g., learning rate, batch size). For reference, our final DeMemSeg model used a ResNet-50 backbone, SGD optimizer, 0.0025 learning rate, and a batch size of 4.

c. Start the training process from the command line inside the Docker container.

3. Evaluate and select the best model

a. Monitor the segm_mAP on the validation set during training by inspecting the generated log files or using visualization tools like TensorBoard if configured.

b. After training is complete, select the model checkpoint (pth file) that provides the best performance based on your chosen metrics (e.g., highest overall segm_mAP or highest segm_mAP_75 for precision).

c. The final trained model checkpoint can then be used for inference as described in section B2.

*Note: The model selection criteria should align with your research goal. For precise shape analysis, prioritizing segm_mAP_75 is recommended, as discussed in the main research article.*

**B. Performing inference on PSMs with the pretrained DeMemSeg model**

This section provides a step-by-step guide for using our pretrained DeMemSeg model to segment PSMs. All necessary code and models are available in our primary GitHub repository: https://github.com/MolCellBiol-tsukuba/DeMemSeg.

**B1. Environment setup**

1. Clone the DeMemSeg GitHub repository and navigate into its directory.

```bash

git clone https://github.com/MolCellBiol-tsukuba/DeMemSeg.git

cd DeMemSeg

```

2. Build and run the Docker Container. Use the provided Dockerfile to create a containerized environment with all required dependencies. This ensures complete reproducibility of the software environment.

```bash

docker build -t dememseg-env:latest . docker build -t dememseg-env:latest .

docker run -it --name dememseg_container --gpus all -v "$(pwd)":/workspace -p 8888:8888 dememseg-env:latest

```

3. Connect to the Container with an IDE. For ease of use in editing files and running commands, we recommend connecting to the running dememseg_container using Visual Studio Code (VS Code) with the Docker extension.

**B2. Running the inference and analysis workflow**

1. Prepare input images: Place your 2D MIP images of PSMs into a designated input folder inside the repository, for example, input_images/.

2. Open the Jupyter Notebook named main_DeMemSeg.ipynb.

3. Specify input and output paths: In the initial cells of the notebook, you must modify the parameters to set the correct paths to your input image folder and your desired output folder. Following the examples provided in the notebook is the recommended way to get started.

4. Execute the notebook cells. Execute all the cells in the notebook. The notebook is designed to automatically perform the entire pipeline:

a. Cell cropping: It first applies the provided custom CellPose model to identify and crop valid single cells.

b. PSM segmentation: It then runs inference with the pretrained DeMemSeg model on each cropped cell image.

c. (Optional) Morphological analysis: Finally, it can calculate morphological parameters from the segmentation masks and generate quantitative plots.

**B3. Locating and interpreting the output**

**Figure 4.**
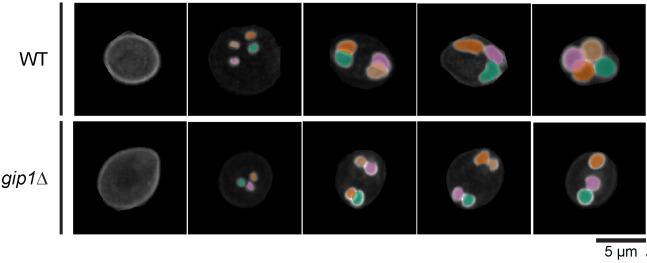
DeMemSeg accurately segments prospore membranes in both wild-type and mutant cells. Representative maximum intensity projection (MIP) images are shown for wild-type (WT; top row) and *gip1*Δ (bottom row) cells expressing the prospore membrane marker mCherry-Spo20^51–91^ shown in gray scale color. The colored overlays represent the individual prospore membrane (PSM) instances as predicted by the DeMemSeg model. Scale bar, 5 μm.

1. Check the output directory. The results of the pipeline, including segmented mask images, JSON files with instance coordinates, and any generated figures or CSV files, will be saved in the output directory you specified in the notebook.

2. Review the results. The generated outputs can be used for your downstream analysis. Refer to Figures 3D, 4, and 5 in [7] as examples of the visual and quantitative results that this pipeline can produce.

*Note: This pretrained model and workflow are optimized for images with characteristics similar to those described in section A2. Performance on significantly different image types may vary (Figure S4 in [7]).*

**B4. Extracting morphological features to a CSV file**

Following instance segmentation by DeMemSeg, a custom Python script leveraging the OpenCV library processes the output JSON files to extract a comprehensive set of morphological parameters from each individual membrane contour. This script automates the entire feature extraction workflow, which consists of the following key actions:

1. Decode masks: The script first reads the RLE (Run-Length Encoded) mask data for each detected object from the input JSON files.

2. Quantify morphological features: From each decoded mask, it then calculates a comprehensive set of parameters. While our biological analysis in this study focused on perimeter and roundness, the full set of extracted features are as follows:

a. Basic geometric properties: Area, perimeter, major axis length, and aspect ratio.

b. Derived shape metrics: Roundness and compactness, which collectively describe the form of the membrane.

c. Advanced shape descriptors: A set of seven translation-, rotation-, and scale-invariant Hu moments, along with Fourier descriptors to characterize the boundary in detail.

d. Positional and intensity data: The x, y coordinates of the centroid and the mean pixel intensity within the segmented region.

3. Compile data: Finally, the script aggregates the quantitative data from all detected objects into a single, well-structured CSV file, with each row corresponding to a single membrane, ready for downstream statistical analysis.

The full Python script used for this feature extraction, detailing all calculated parameters, is publicly available on our GitHub repository to ensure full reproducibility of our analysis pipeline: ( https://github.com/MolCellBiol-tsukuba/DeMemSeg-train/blob/main/script/mmdetection_psm.py ).

**Result interpretation**

The primary output of the DeMemSeg pipeline is a set of instance segmentation masks, typically saved as image files or within a JSON file, for each analyzed cell. Each mask delineates an individual prospore membrane. Successful segmentation is characterized by smooth, continuous boundaries that accurately outline the fluorescently labeled membrane structures, as seen in the examples in **Figure 4**.

**Figure 5.**
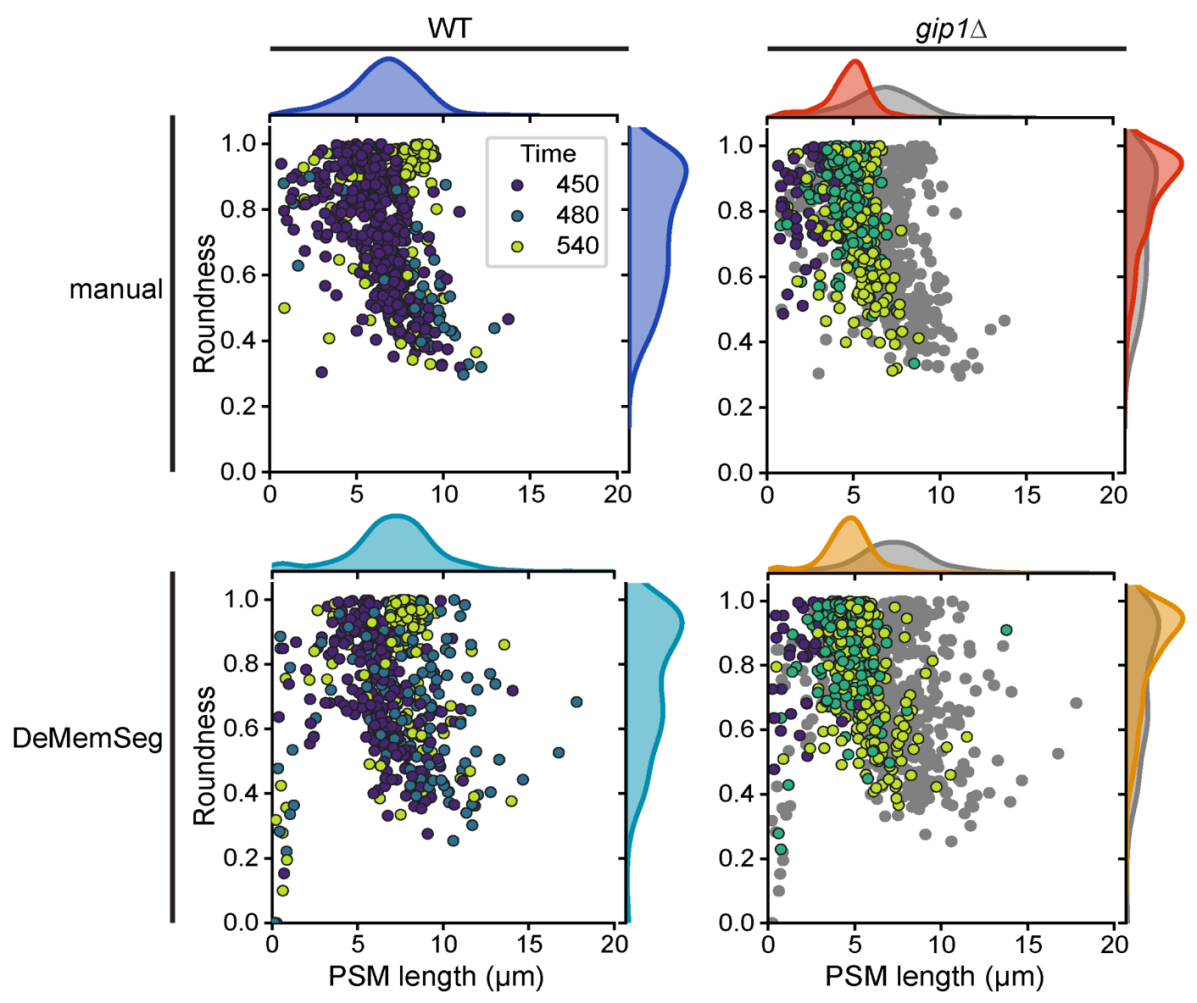
DeMemSeg provides quantitative measurements of prospore membrane (PSM) morphology that are comparable to manual annotation. Scatterplots comparing PSM roundness vs. length for wild-type (WT; left panels) and *gip1*∆ mutant (right panels) cells. The top row shows the results from manual annotation, while the bottom row shows the results from the automated DeMemSeg pipeline. Data points are colored by time (in minutes) after sporulation induction. For the *gip1*∆ column, WT data is also presented as gray points for comparison. The marginal density plots illustrate the overall distribution of PSM length (top) and roundness (right) for each condition.

The downstream analysis scripts generate quantitative morphological data for each segmented PSM, including its length (perimeter) and roundness. These parameters can be interpreted to classify the developmental stage and morphology of the PSMs:

1. Early/dot stage: Characterized by a short perimeter and intermediate-to-high roundness.

2. Horseshoe/elongating stage: Characterized by a long perimeter and low roundness values.

3. Mature/sphere stage: Characterized by a moderately long perimeter and a roundness value approaching 1.0, indicating a spherical shape (**Figure 5**).

The power of this protocol lies in comparing the distribution of these morphological parameters between different experimental conditions, such as wild type (WT) versus a mutant strain (Figure 4). For example, in our validation study using the *gip1*Δ mutant, we used this comparative analysis to draw biological conclusions. By plotting PSM length vs. roundness for both WT and *gip1*Δ populations (Figure 5), a clear shift in the data distribution becomes apparent. The *gip1*Δ mutant population shows a scarcity of data points in the elongating stage region (long perimeter, low roundness), which can be interpreted as a quantitative indicator of a membrane elongation defect. Furthermore, the mutant's failure to populate the mature/sphere stage region (roundness near 1.0) signifies an impairment in the final closure and maturation steps. By interpreting these shifts in the morphological landscape, researchers can quantitatively characterize the effects of genetic mutations or experimental treatments on membrane dynamics.

## Validation of protocol

This protocol and its core components have been used and validated in the following research article:

- Taguchi et al. [7]. Deep learning-based segmentation of 2D projection-derived overlapping prospore membrane in yeast. *Cell Struct Funct*.

The robustness and reliability of this protocol are demonstrated by several key findings presented in the research article. Following systematic optimization, the final DeMemSeg model achieved strong quantitative performance metrics, including a segm_mAP of 0.670 and a segm_mAP_75 of 0.786, with visual validation confirming high individual IoU scores for representative instances (Figure 3 in [7]). Furthermore, the protocol's output was directly compared against the gold standard of manual expert annotation. This comparison revealed no statistically significant differences between morphological measurements (PSM length and roundness) derived from DeMemSeg’s automated segmentation and those from manual annotation for both WT and *gip1*∆ mutant cells (Figure 5B in [7]). Finally, the protocol's practical utility and generalization capabilities were confirmed by its successful application to two types of unseen data: dynamic time-lapse image sequences (Figure 4 in [7]) and the morphologically distinct *gip1*∆ mutant, which was not part of the training data. The ability to quantitatively distinguish the mutant phenotype from the WT population (** Figure 5 **) confirms that the protocol produces reliable and biologically meaningful results.

## General notes and troubleshooting

**General notes**

1. Applicability and adaptability of the workflow: This protocol is modular. The pretrained DeMemSeg model can be directly applied to segment PSMs from images acquired on different microscope systems, provided the input images are first processed into the required single-cell format. To achieve this, users would need to follow the initial steps of the pipeline: train their own custom CellPose model tailored to their specific imaging data to perform whole-cell segmentation, and then use it to generate the cropped, single-cell extract_image files (as detailed in steps A3.1–A3.2). Once these standardized input images are prepared, the PSM segmentation part of the workflow (section B) can be run directly. Furthermore, the entire protocol detailed in section A serves as a general framework that can be adapted to train new models for segmenting other challenging, overlapping subcellular structures in various cell types.

2. Balancing annotation effort and model performance: Manual annotation is the most time-consuming step in this protocol. Our optimization results (Figure 3A in [7]) can serve as a guideline for balancing annotation effort against desired model accuracy. While performance generally improves with more data, we found that a substantial level of performance (segm_mAP > 0.65) was achieved with approximately 3,500 annotated PSM instances (50% of our full dataset). Researchers adapting this protocol for new tasks can use this as a benchmark to estimate the amount of annotation required to achieve a robust initial model.

3. Key sources of variability: The primary source of variability that can affect model performance is the consistency of manual annotation. Subtle differences in how boundaries are traced, especially between different annotators, can introduce noise into the training data. To minimize this, we recommend that a single person annotates the entire dataset if possible, or that a very clear, shared annotation guideline is established and followed by all annotators. A second source of variability is image quality; significant fluctuations in signal-to-noise ratio, illumination, or focus across the dataset can impact the performance of both the CellPose and DeMemSeg models. Maintaining consistent imaging conditions is highly recommended.

**Troubleshooting**

**Problem 1:** The model generates false positives by segmenting prospore membranes in cells adjacent to the central target cell.

Possible cause: This issue typically arises if the model is trained on, or asked to perform inference on, images that contain more than one cell. Deep learning models analyze all the visual information present in an input image. The crop_image generated in the initial cropping step is a simple square cut-out and may still contain fragments of neighboring cells. If the model sees these fragments, it may correctly identify them as PSM, leading to segmentations outside the cell of interest, which are considered false positives in the context of single-cell analysis.

Solution: The solution is to use the cell-extracted image for both training and inference. As detailed in **step A3.2**, the cell-extracted image is created by applying the CellPose-generated whole-cell mask to the crop_image. This process effectively erases all visual information outside the single, central target cell. By training the model exclusively on these "cleaned" single-cell images, you teach it to focus only on the structures within the correct cell boundary. Consistently using extract_image files for both training and final analysis is a critical step to prevent this type of false positive detection.

**Problem 2:** A single, identical PSM is detected multiple times, resulting in over-detection with several overlapping masks for the same object. This can be more common when lowering the prediction score threshold to capture faint objects.

Possible cause: Deep learning models assign a "prediction score" to every potential object they find. To obtain the final results, we set a score threshold; predictions above this threshold are kept, while those below are discarded. If this threshold is set low, the model might keep several slightly different but highly overlapping predictions for the same object, all of which have scores above the low threshold. This results in the over-detection of a single PSM.

Solution: The most direct solution is to leverage biological context by setting the maximum number of instances to detect per image (max_per_img parameter). Since a single sporulating yeast cell produces a maximum of four spores, and thus four PSMs, we can set this parameter to 4. This instructs the model to "No matter how many potential objects you find in this image, only return the top 4 with the highest prediction scores." This effectively filters out lower-scoring duplicate detections and prevents biologically impossible outcomes. This parameter is configured during the inference step.

**Problem 3:** When two PSMs are heavily overlapping, the model fails to detect one of them, segmenting only the one with the higher prediction score.

Possible cause: This problem is often caused by a post-processing step called non-maximum suppression (NMS). NMS is essential for cleaning up redundant predictions. It works by comparing the intersection over union (IoU) of any two predicted masks. If the IoU value is above a set threshold, NMS assumes the two predictions are just duplicates of the same object and discards the one with the lower prediction score. The default NMS IoU threshold in the MMdetection framework is 0.5. However, two real, distinct PSMs can be so close that their masks overlap by more than 50% (IoU > 0.5). In this case, the default NMS setting will incorrectly treat them as duplicates and suppress one, leading to an under-detection.

Solution: The solution is to increase the NMS IoU threshold to a value that is higher than the typical IoU of two distinct but heavily overlapping PSMs in your dataset. By raising the threshold (in our case, to 0.8), we are telling the algorithm to "Only consider two predictions to be duplicates if they are extremely similar (more than 80% overlap)." This adjustment allows the model to correctly identify two distinct PSMs that might have, for example, 60% or 70% overlap as separate instances. This is a critical parameter to tune using a validation dataset, especially when working with objects that are known to be densely packed or overlapping.
